# Soil CO_2_ emission in response to organic amendments, temperature, and rainfall

**DOI:** 10.1038/s41598-020-62267-6

**Published:** 2020-04-03

**Authors:** Ram L. Ray, Richard W. Griffin, Ali Fares, Almoutaz Elhassan, Ripendra Awal, Selamawit Woldesenbet, Eric Risch

**Affiliations:** Cooperative Agricultural Research Center, College of Agriculture and Human Sciences, Prairie View A&M University, Prairie View, TX 77446 USA

**Keywords:** Climate sciences, Ecology, Environmental sciences, Hydrology

## Abstract

Vegetated land surfaces play an important role in determining the fate of carbon in the global carbon cycle. However, our understanding of the terrestrial biosphere on a global scale is subject to considerable uncertainty, especially concerning the impacts of climatic variables on the carbon cycle. Soil is a source and also a sink of CO_2_ exchange and helps in carbon sequestration. Agricultural management practices influence soil water dynamics, as well as carbon cycling by changing soil CO_2_ emission and uptake rates. The rate of soil CO_2_ emission varies for different crops and different organic amendments. The major goal of this study was to assess the impacts of the type and rate of organic amendment on soil CO_2_ emission in a collard greens crop grown in the southeast Texas environment. Thirty-six plots were developed to grow collard greens on Prairie View A&M University’s Research Farm. Three types of organic amendments (Chicken manure, Dairy manure, and Milorganite), at four levels of application (0, 168, 336, and 672 kg N/ha) were used and replicated three times. Each organic amendment type was applied to nine randomly selected plots. Three random plots were used as a control in each row. We measured daily soil CO_2_ emission for the first two weeks and every other day in a week during the experiment. We evaluated the effects of organic amendments and the application rates on soil CO_2_ emission for collard greens during two growing seasons. The results showed higher the application rates for each organic amendment, higher the CO_2_ emissions from the soil. The results also showed higher cumulative CO_2_ emissions for the soils amended with chicken manure and milorganite, but lowest for the soils amended with dairy manure. This field experiment and analyses help better understand the temporal and spatial variations of soil CO_2_ emission, and also help to develop best management practices to maximize carbon sequestration and to minimize soil CO_2_ emissions during the growth periods of collard greens under changing temperatures using different organic amendments, and application rates.

## Introduction

Vegetated land surfaces play a significant role in controlling the carbon dynamics in the global carbon cycle; however, knowledge about the comprehensive role of the terrestrial biosphere on regional to global scale under changing climate is still limited^1^. Greenhouse gases, including CO_2_ emissions, are rapidly increasing because Earth’s climate is continuously warming^2–4^. The physiological processes of vegetation leaves and photosynthetic capacity, which influence carbon emission and carbon uptake, rely on daily and seasonal variations of weather parameters, and hydrologic and climatic variables (e.g., solar energy, soil and air temperatures, humidity deficits, soil moisture)^5–10^. Hence, CO_2_ fluxes over agricultural lands are expected to vary on daily and seasonal time scales.

During the growing season, vegetation coverage reduces bare soil areas to the environment. For example, in the beginning, the soil surface used to be fully exposed to the atmosphere, which gradually decreased along with the vegetation growth. The bare soil, which gradually covered with moss, would also have an impact on S_CO2_ emission. Several physical, chemical, and biological properties of soils, climatic parameters (e.g., temperature, and rainfall), and hydrologic parameters (e.g., soil moisture) are responsible for characterizing spatial and temporal variability of S_CO2_ emission. In addition, agricultural activities and management practices also influence S_CO2_ emissions because they can alter soil organic matter or soil carbon^11,12^.

Terrestrial carbon sequestration plays an important role in increasing the concentration of atmospheric CO_2_^13,14^. Atmospheric CO_2_, one of the major greenhouse gases, which has an impact on global warming, and its variation is in response to global natural and human activities. Changes in climate, such as precipitation and temperature, could alter the exchanges of these gases by altering plant activities, as well as thermal and hydrologic regimes^15^.

An ecosystem can be a net carbon source or sink; however, it is necessary to evaluate the long-term carbon flux dynamics in order to understand the movement of carbon into and out of an ecosystem^16^. The previous study suggests that ecosystems (e.g., grasslands, agricultural lands) may act as carbon sinks in one year and then act as carbon sources another year, because of independent and different variations in photosynthesis and respiration^17^. It is understood that even a small change in the duration or extent of the crop growing season could result in large changes in annual CO_2_ flux^18^. During the growing season, daily CO_2_ fluxes display characteristic spatial patterns of positive and negative fluxes at the synoptic scale^19^. Currently, since the global climate is changing, more attention is paid to reduce greenhouse gases emission to maintain a sustainable environment^19,20^. However, it is also important to monitor CO_2_ emission and uptake worldwide to achieve a sustainable environment^21,22^.

Soil processes play important roles in global climate change as soils have the potential to act as a net sink for CO_2_ due to a large amount of carbon currently stored in soil organic matter^23–26^. Soils are major sources of CO_2,_ and some agricultural soils have large potential for soil carbon sequestration and atmospheric carbon reduction through soil CO_2_ flux^27,28^. However, agricultural management practices play a critical role in enhancing soil carbon sequestration. Agricultural management practices that increase the input of organic matter and decrease the soil organic matter decomposition rates help build up soil carbon^29,30^. On the other hand, soil CO_2_ emissions include the respiration of soil organisms and plant roots^28^.

Soil organic amendments are essential for sustainable agriculture because they help to improve plant growth, crop yield, soil carbon content, and microbial biomass and activity^31,32^. Organic amendments affect the rate and extent of soil carbon sequestration; however, they have some disadvantages such as eutrophication of surface waters and greenhouse gas emission (GHG)^32,33^. Investigating the impacts of type and rate of organic amendments on GHG emission is thus important for sustainable agriculture and minimizing the impacts on the GHG emission.

Numerous studies have investigated the response of carbon and other GHG emissions with and without organic amendments. For example, Zhang *et al*.^34^ evaluated the effect of biochar amendment (three different rates) on yield and methane and N_2_O emission from a rice paddy in China. They found 12–14% higher rice yield and 34–41% higher CH_4_-C emission as a result of the organic amendment. Álvaro-Fuentes *et al*.^35^ studied the impact of organic amendment and tillage management practices on soil CO_2_ flux and found that tillage and fertilization significantly impacted soil CO_2_ fluxes. Amos *et al*.^36^ studied fertilizer impact on irrigated Maize and documented a 64% CO_2_ flux increase compared to the control treatment.

Davidson *et al*.^37^ examined differences in soil CO_2_ emissions among primary forests, secondary forests, active cattle pastures, and degraded cattle pastures, and found the rates of soil respiration decreased from wet to dry seasons in all land uses. Melling *et al*.^24^ measured monthly soil CO_2_ emission from three ecosystems; forest, sago, and oil palm, and found a significant impact of land use on the exchange of CO_2_. Abbas and Fares^38^ evaluated impact of rates and types of organic amendment on soil organic carbon and CO_2_ emissions under a sweet corn and a tropical soil; they found that soil organic carbon content, CO_2_ emissions, and saturated hydraulic conductivity of the soil significantly increased with addition of organic amendment and/or increase in organic amendment rates, but no significant effect of organic amendment types.

Few studies have been conducted to monitor soil organic carbon and CO_2_ emission for leafy greens^39,40^. Ingram *et al*.^39^ investigated the effects of repeated irrigation on total organic carbon on baby spinach. Tavarini *et al*.^40^ studied the effects of green compost on soil biochemical characteristics and nutritive quality of leafy vegetables. On the other hand, limited research has been conducted on collard greens in the southeast Texas environment e.g.^41–43^.

The effects of organic amendments on soil organic carbon and CO_2_ emission have received little attention from researchers. Most studies have focused on the impact of organic amendments on crop yield, soil characteristics, and soil nutrition^43^. After a comprehensive literature review, we determined that few studies have been conducted to examine the impact of types and rates of organic amendments on soil organic carbon content and CO_2_ emission under a collard greens crop grown in the southeast Texas environment.

The primary goal of this research work was to assess the impacts of organic amendment types and rates on the soil CO_2_ emission under a collard greens grown in the humid southeast Texas ecosystem. Specific objectives were to quantify: (i) the effect of organic amendment types and application rates on soil CO_2_ emission from a Wockley fine sandy loam soil; and (ii) the relationship between soil CO_2_ emission, soil organic carbon, temperature, and rainfall.

## Materials and Methods

### Site descriptions

**Figure 1:**
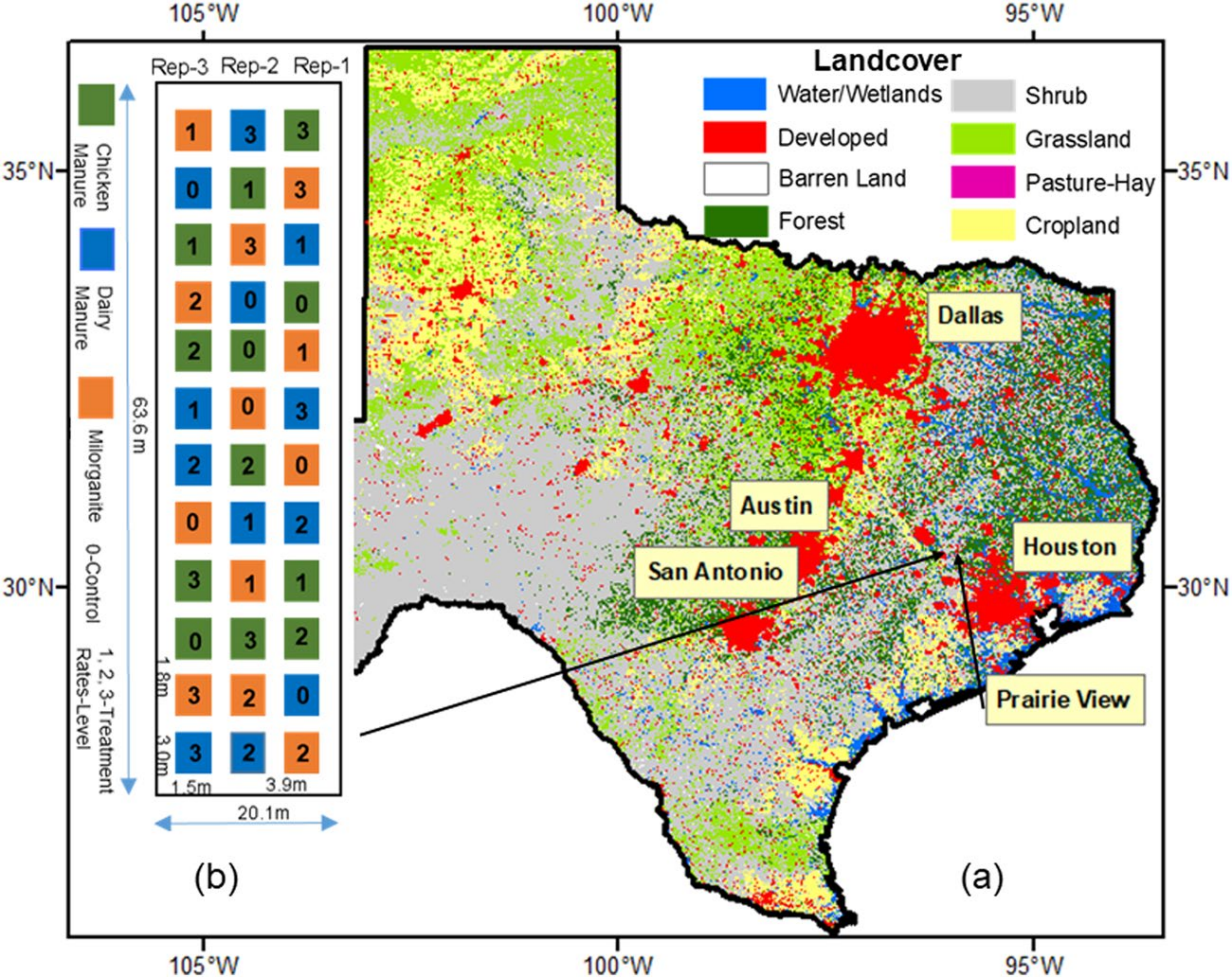
Experimental site was at the Research Farm of Prairie View A&M University, Prairie View, Texas (**a**). Figure b, on the left, is the experimental layout with thirty-six plots. (Landcover data source: National Land Cover Database (NLCD))^54^.

The study was conducted on the Research Farm of the College of Agriculture and Human Sciences (CAHS) of Prairie View A&M University (PVAMU), Prairie View, Texas (Fig. 1). PVAMU is located northwest of the Greater Houston Metropolitan Area with an average annual rainfall of about 1,118 mm yr^–1^ (based on 1981–2010 data), with over 60% occurring between June and October. The climate is hot during summer and cooler during winter. July is the hottest month with an average air temperature of 35 °C, while January is coldest with an average air temperature of 3 °C. Daily precipitation and temperature data were measured from an installed weather station located in the middle of the experimental plots. The soil at the site is classified as a Wockley fine sandy loam.

### Experimental design

The effects of organic amendment types and their application rates on CO_2_ emission were tested using completely randomly selected plots on the research farm of PVAMU. The combination of three organic amendment types (chicken manure, dairy manure, and milorganite) and three rates (low, medium, and high) and a control (no amendment) were randomly replicated three times. Each plot was 1.52 m wide and 3.05 m long. The longitudinal and lateral spacing of the plot was 1.8 m and 3.9 m, respectively (Fig. 1). The bed of each plot was raised by 30 cm for proper drainage. Organic amendments were manually incorporated into the soil. Amendment application rates were calculated on a dry weight basis based on equivalent nitrogen (N) contents of 3, 0.5, and 5% for chicken manure, dairy manure, and milorganite, respectively (Table 1). Selected chemical properties of chicken manure, dairy manure, and milorganite are given in Table 2. Target N application rates of 168, 336 and 672 kg N ha^−1^ representing low, medium and high rates, respectively, were used based on the recommendations from previous researchers^38,44^.

**Table 1:** The Organic amendment weights and their macro-element composition for different rates.

Manure Types	Treatment Rates (kg- ha^−1^)			
	Level	1	2	3
	0	168	336	672
Chicken Manure (3%N, 2%P, 3%K)	0	2.6	5.2	10.4
Dairy Manure (0.5%N, 0.5%P, 0.5%K)	0	15.6	31.2	62.4
Milorganite, (5%N, 4%P, 0%K)	0	1.6	3.1	6.2

**Table 2:** Macro and micro-chemical elements of three organic amendments.

Chemical Elements	Chicken Manure	Dairy Manure	Milor-ganite	Chemical Elements	Chicken Manure	Dairy Manure	Milor-ganite
	g kg^−1^	g kg^−1^	g kg^−1^		g kg^−1^	g kg^−1^	g kg^−1^
Nitrogen	30 (3%)	2(2%)	50 (5%)	Calcium	10.63	9.83	6.14
Carbon	317.3	84.25	338.14	Iron	0.26	0.16	2.15
Hydrogen	53.5	9.67	57.53	Magnesium	3.90	0.36	2.17
Sulphur	10.00	1.20	8.88	Manganese	0.27	0.07	0.52
pH Level (1–14)*	7.8	8.16	6.06	Phosphorous	5.51	0.29	2.41
EC (ms/cm)**	5.81	3.43	3.84	Potassium	24.01	0.54	1.54
Aluminum	0.36	0.41	0.63	Sodium	5.56	0.45	0.85
Boron	0.28	0.07	0.10				

(*pH scale 1–14 and **EC unit mS cm^−1^).

The chemical properties and composition of the three organic amendments were analytically determined in the laboratory. Dairy manure has the highest pH (8.16), whereas milorganite had the lowest pH (6.06). The highest (5.51, 24.01, and 5.56 g kg^−1^) and lowest (0.29, 0.54, and 0.45 g kg^−1^) P, K, and Na were observed in chicken and dairy manure, respectively (Table 2). On the other hand, the highest and lowest total soil carbon emissions were observed in milorganite and dairy manure, respectively.

**Figure 2:**
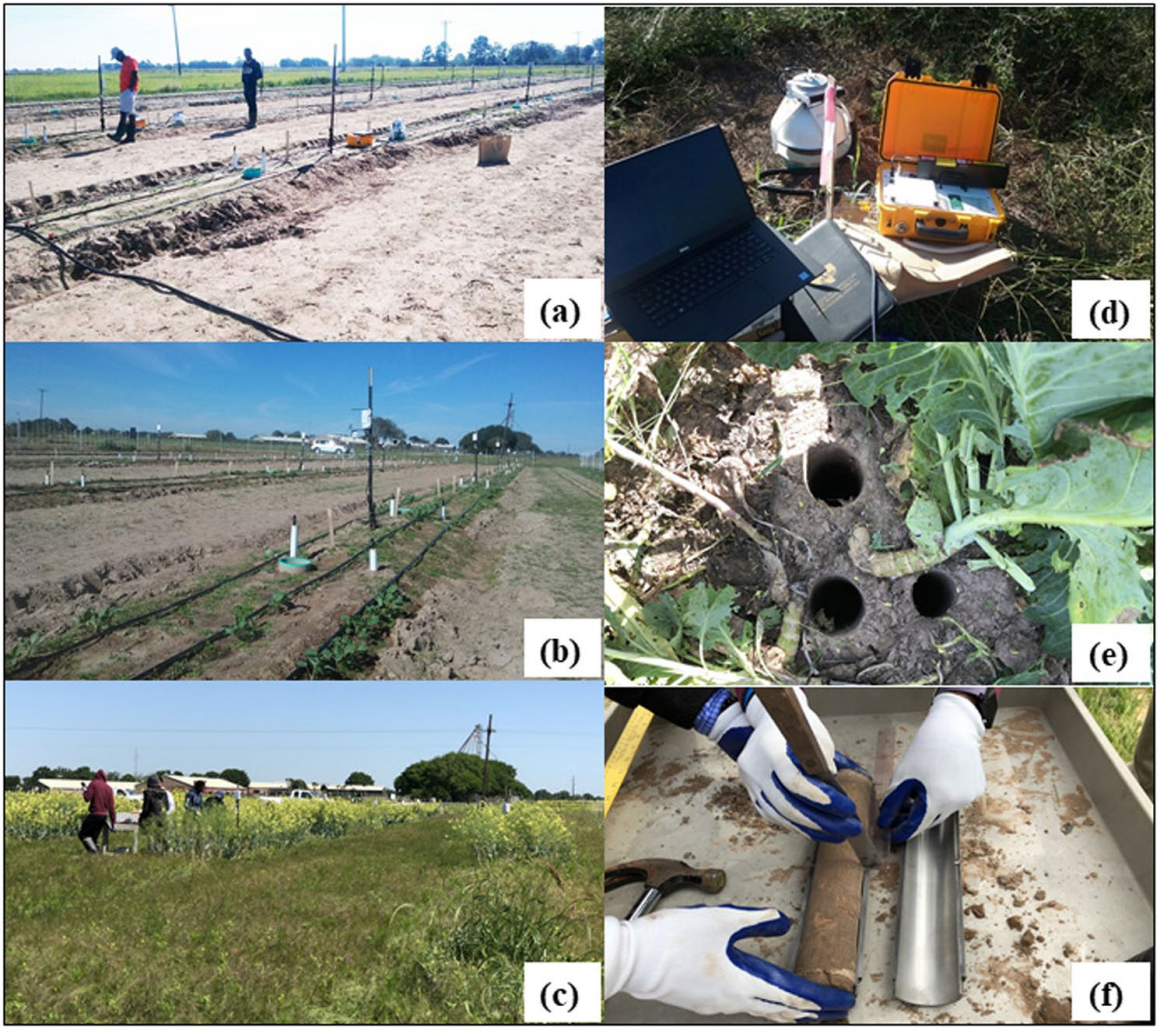
A view of the experiment site: within a few days (**a**), after several weeks (**b**), and (**c**) a few months after its start. (**d**) LI-COR carbon-dioxide sampling system, (**e**) locations of soil samples that were used in soil physical, chemical and organic carbon content analyses, and (**f**) processing of soil sample samples collected in the upper part (0 to 15 cm) and the lower part (16 to 30 cm) of the root zone.

The four treatments included; one with no fertilizer as a control and three fertilizer application rates; 168 kg N ha^−1^, (half of the recommended N rate), 336 kg N ha^−1^ (recommended N rate); and 672 kg N ha^−1^ (double of recommended rate). The manure application rates were based on their equivalent nitrogen (N) content. The Organic amendment weights and their macro-element composition for different rates used are presented in Table 1. The experimental field was adequately prepared before the surface broadcasting of the organic amendments. The organic amendments were thoroughly mixed to ensure appropriate incorporation in the root zone. During the first growing season, collard greens was sown on October 25, 2017, and the first harvest was on March 31, 2018, whereas, during the second growing season, collard greens was sown on November 20, 2018, and harvested on April 30, 2019. The carbon dioxide emission measurements started immediately after sowing and continued until May 30, 2019 (Fig. 2a–c). The cropped area was irrigated using drip irrigation method as deemed necessary. Four irrigation events (13^th^, 20^th^, 24^th^ of November and 1^st^ of December in 2017 in growing season 1 and 23^rd^, 26^th^ of April and 1^st^, and 29^th^ of May 2019 in growing season 2) were scheduled to meet the crop’s water demand based on rainfall event and crop growth stage. There were adequate rainfall events during the first few months during the second growing season (November 2018 to March 2019) and during the last few months during the first growing season (January to March 2018); as such there was no need to schedule any supplemental irrigation events.

### Measurement of soil CO_2_ flux

A soil collar, suitable for a 20 cm survey chamber, was installed at each plot. Soil carbon dioxide CO_2_ (S_CO2_) emission was measured using a LI-8100A (LI-COR Biosciences, Lincoln, NE) survey system before and after the application of the organic amendments at each plot of the experiment during the two growing seasons. The survey system includes a data logger, survey chamber, and soil collar. LI-COR Biosciences proprietary Soil-Flux-Pro software was used to operate the system and analyze S_CO2_ flux data logged by the data logger (Fig. 2d).

S_CO2_ emission was measured daily for the first two weeks, and every other day during the rest of the growing season. The times of measurement were between 9:00, and 11:00 am during the length of the experiment. The selected time for measurements is considered optimal sampling time to represent the average daily S_CO2_ emission^45,46^.

### Soil sampling and analysis

Soil samples were collected with a soil core sampler (diameter 5 cm and height 30 cm) at three locations around a selected collard greens plant (Fig. 2e) per plot. Soil samples were collected at the end of the growing season from each plot from the top 30 cm soil and separated into 0–15 cm and 16–30 cm layers (Fig. 2f). The soil samples were air-dried. Roots, fauna, and organic debris were removed before soil samples were pulverized and sieved through a 2 mm soil sieve in the Cooperative Agricultural Research Center-Core Laboratory. The samples were later stored at room temperature before analyzing for carbon and pH. Air-dried soil samples (<2 mm) were used to determine soil pH from a 1:2.5 (w/v) mixture of soil and water. A portion of the air-dried samples was ground (<0.25 mm) prior to the total carbon and soil organic carbon analyses. Total soil carbon content was determined using Elementar Vario Macro Cube CHNS Analyzer (Vario EL III, Elementar, Germany) and following a standard methodology as detailed in Pallasser *et al*.^47^. A 150–155 mg of dry soil was placed in a tin foil capsules and combusted in the Elementar. During the combustion process, temperatures of the combustion tube and reduction tube were maintained at 1100 °C and 850 °C, respectively. For soil organic carbon, the process was similar to total soil carbon, except the samples were treated with sufficient phosphoric acid (1:1) to remove inorganic carbon prior to instrument analysis, which was combusted at 1,100 °C.

### Data analysis and method

Daily average CO_2_ emission rates were calculated for each of the 36 experimental plots. ArcGIS tool was used to develop spatial distribution maps of S_CO2_ emission for selected dates; before the treatment (October 10, 2017), after the treatment (October 31, 2017), during the treatment (February 5, 2018), and at harvesting (March 30, 2018) in the first season, and after the treatment (November 28, 2018), during the treatment (February 5, and March 06, 2019), at harvesting (April 30, 2019), and after harvesting (May 30, 2019) in the second season. First, S_CO2_ emission of each treatment type and application rate were compared with the control treatment. Second, S_CO2_ emissions of normal application rates of each chicken manure, dairy manure, and milorganite treatments were compared with their low and high application rates to understand the variability among them. Temporal distributions of S_CO2_ emission were also compared with the temporal variations of rainfall, air temperature, and soil temperatures at 5-cm and 50-cm, respectively. Third, a SigmaPlot tool was used to develop box and whisker plots for each treatment type and application rate. Finally, S_CO2_ emission data were analyzed using analysis of variance (ANOVA) to estimate the coefficient of determination (R^2^), p-value, and root mean square of error (RMSE) to characterize the differences among treatments and application rates.

## Results

### Effects of organic amendments on the temporal variation of soil CO_2_ emissions

**Figure 3:**
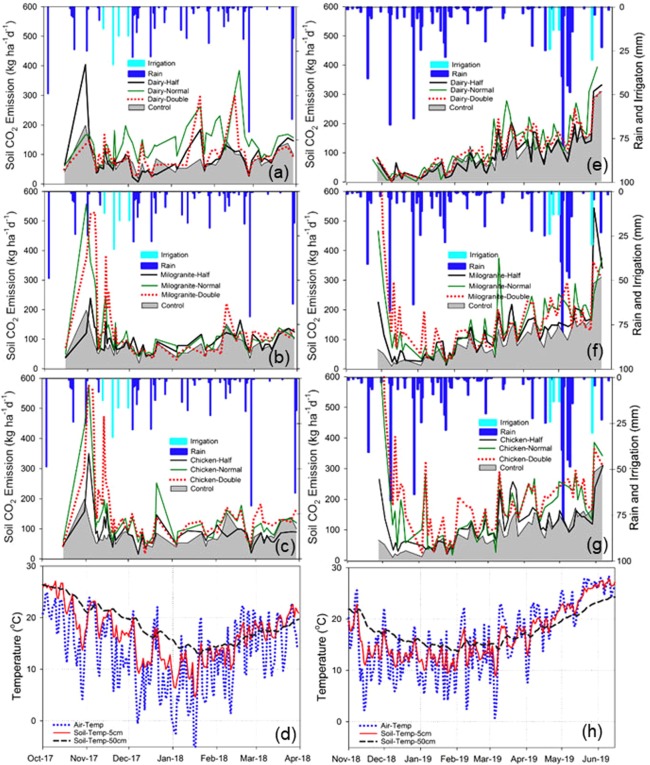
Temporal distribution of soil carbon dioxide emissions, rainfall and air and soil temperatures during the two growing seasons. Figure 3a–c,e–g compare daily rainfall, CO_2_ emission from control and soils amended with dairy manure, chicken manure, and milorganite, respectively, in growing season 1 and 2. Figure 3d,h compare air temperature and soil temperatures at 5 and 50 cm soil depths, respectively, in growing seasons 1 and 2.

Regardless of organic amendment types and their application rates, S_CO2_ emission spiked following the organic amendment and then gradually decreased; it reached the lowest levels in three months after the start of the experiment (Fig. 3). Figure 3(a–c) depicts S_CO2_ emissions, rainfall, and applied irrigation for three organic amendments types, application rates, and control during the study period in growing season 1 (2017–2018), whereas Fig. 3(e–g) depicts the same data for the same treatments during the growing season 2 (2018–2019). A comparison of air and soil temperatures at 5 cm and 50 cm depth during the growing seasons 1 and 2 are detailed in Fig. 3d,h, respectively.

**Figure 4:**
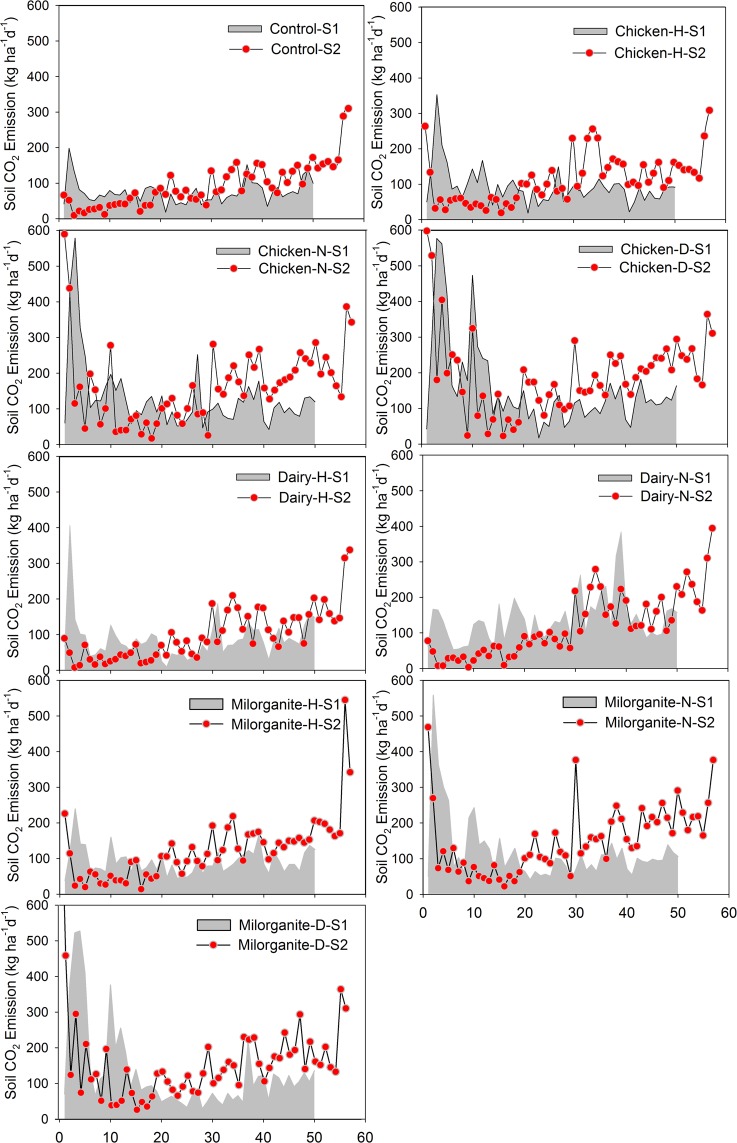
Comparison of soil CO_2_ emissions during growing season 1 and 2 from sowing to harvesting (x-axis in days) to evaluate the impact of three organic amendments and their corresponding application rates (n = 50 days for growing season 1 (S1) and n = 57 days for growing season 2 (S2)). *Note: N* = *Normal, H* = *Half, and D* = *Double application rates*.

Except for the dairy amendment, both milorganite and chicken amendments showed high S_CO2_ emission rates following the organic amendment application. On the other hand, medium and high application rate treatments released more CO_2_ before harvesting compared to the low application rate treatments. High S_CO2_ emission continued for a week before it started to decrease. Amended treatments had higher S_CO2_ emissions than the control treatment during both growing seasons. However, soil temperature and rainfall significantly impacted S_CO2_ spatio-temporal emissions; S_CO2_ emissions were elevated for amended and controlled plots after each rainfall event and under warm soil conditions. Similar amounts of rainfall were received during the two growing seasons (487 mm-Season 1 and 464 mm-Season 2); however, only season 2 showed an increasing trend in S_CO2_ emission from sowing to harvesting. There were similar emission rates during the first week of the two growing seasons; however, S_CO2_ emission rates were consistently higher throughout the second growing season (Fig. 4).

### Effects of organic amendments on the spatial variation of soil CO_2_ emissions

**Figure 5:**
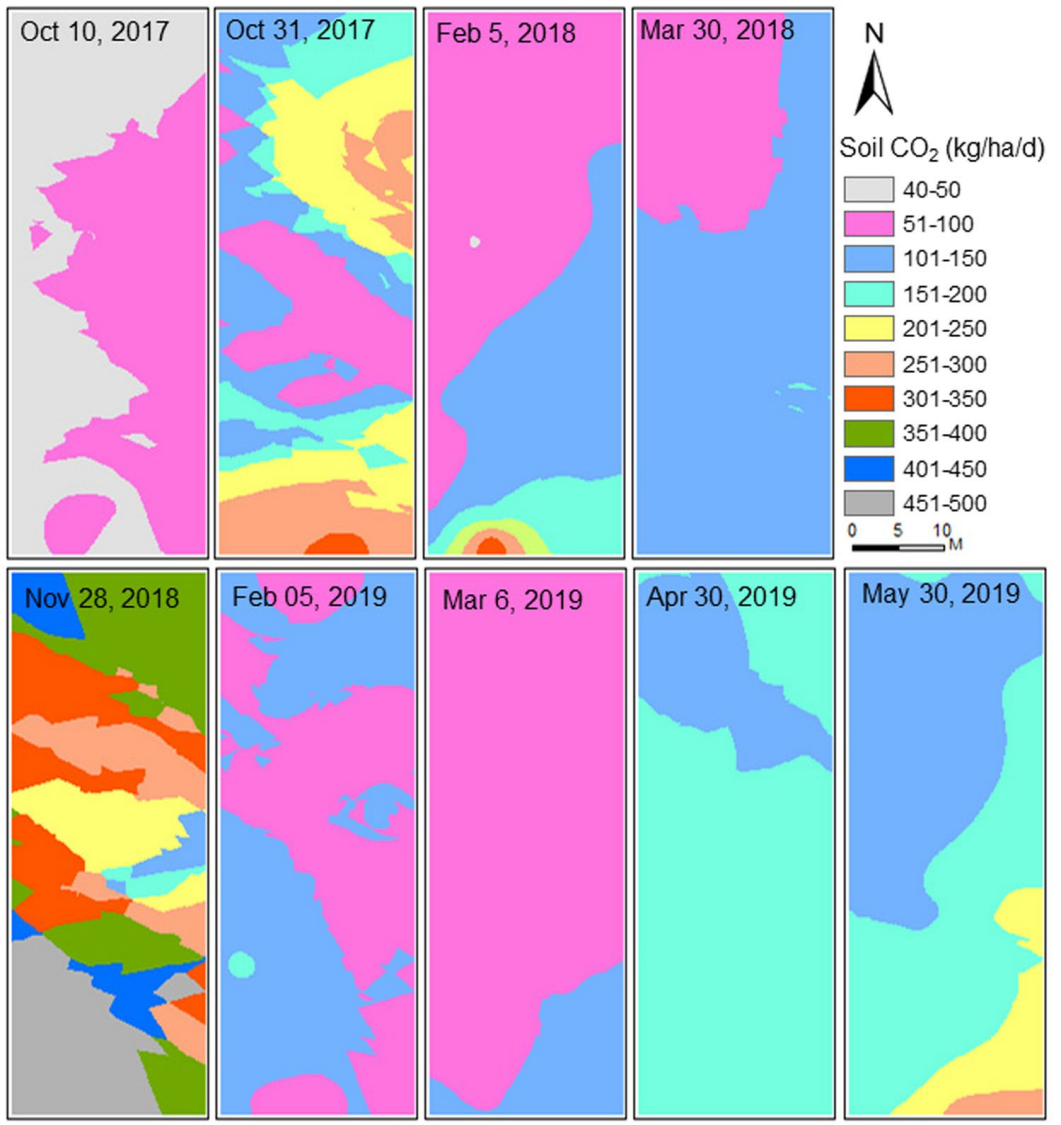
Spatial distribution of soil carbon emissions before and after the application of the organic amendment and during the growing seasons.

Spatial variability of S_CO2_ emission was analyzed for selected dates focusing on before and after plantings, harvesting, and during the growing seasons (Fig. 5). The area within the collar, where the CO_2_ was measured, was kept the plant free to eliminate any plant respiration impact on measured CO_2_ emission at each plot. Although the experimental plots might occupy relatively small areas, spatial distribution maps showed high spatial variability in S_CO2_ emission during the two growing seasons. However, high spatial variability of S_CO2_ emission was observed during the week when amendments were applied during the two growing seasons (Fig. 5). Results showed high spatial variability after the amendment application on October 31, 2017 (growing Season 1) and November 28, 2018 (growing Season 2). After the amendment application, the impact of organic amendment types and their application rates on S_CO2_ emission were high. However, season 2 showed higher (101–500 kg ha^−1^ day^−1^) S_CO2_ emission rates than season 1(51–350 kg ha^−1^ day^−1^). On the other hand, both seasons showed a gradual decrease in S_CO2_ emission after the amendment application.

The range of S_CO2_ emission on February 5 of 2018 and 2019 was different, but most of the study areas had S_CO2_ emission between 51 and 150 kg ha^−1^ day^−1^ in two different growing seasons. During growing season 1, the southern portion of the study area showed slightly higher S_CO2_ emission, which was reduced after February 2018 when the air temperature increased in March 2018. Similarly, during growing season 2, S_CO2_ emission gradually decreased until March 2019, but gradually increased between March and May 30, 2019, which was our last measurement of the season. For example, the observed range of S_CO2_ emission ranged from 51 to 150 kg ha^−1^ day^−1^ in March 2019 and 101 to 200 kg ha^−1^ day^−1^ in April, which was increased to 101 to 300 kg ha^−1^ day^−1^ by May 30, 2019.

### Effects of organic amendment types and application rates on soil CO_2_ emissions

**Figure 6:**
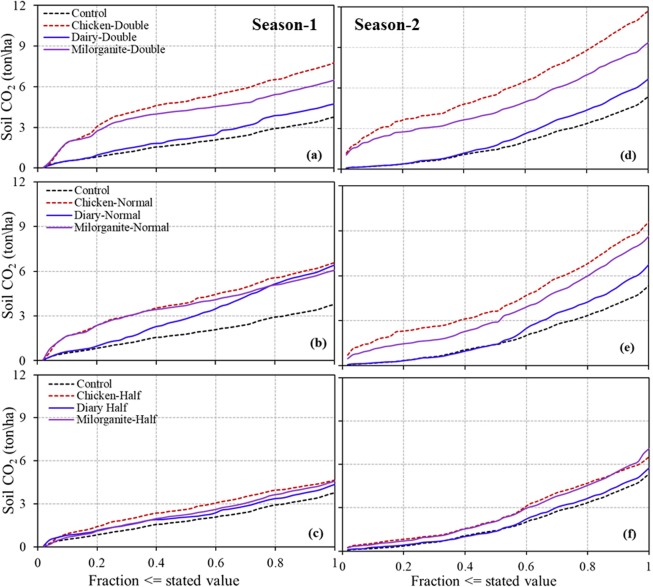
Cumulative soil CO_2_ emissions in growing seasons 1 and 2 with three organic amendments and three application rates (n = 50 days for growing season 1 and n = 57 days for growing season 2).

**Figure 7:**
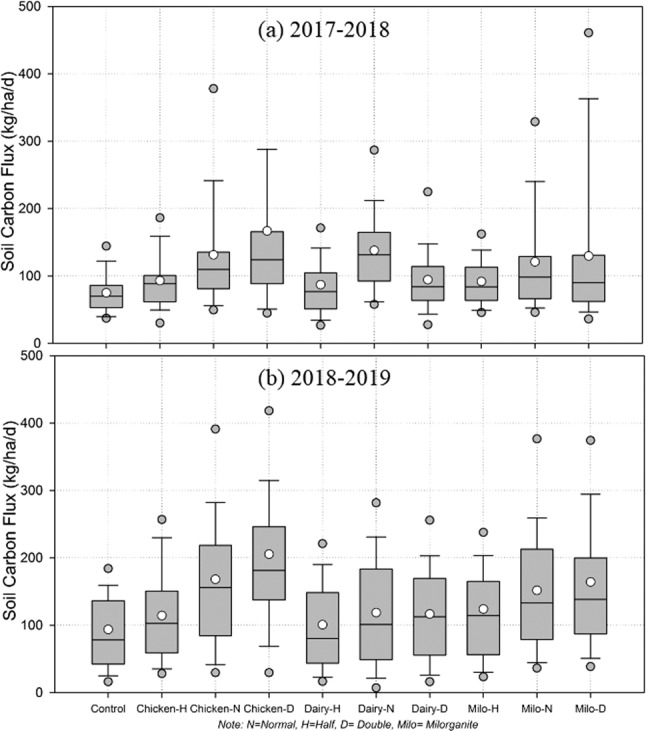
Box and Whisker plots of soil carbon flux from collard greens farm with three different rates and types of organic amendments; (**a**) growing season 1 (2017–2018), and (**b**) growing season 2 (2018–2019). Each boxplot presents mean (white circle), median, 25%, and 75% percentiles, and outliers (in a grey circle), describing the distributions of soil CO_2_ during the growing seasons (n = 50 days for season 1 and n = 46 days for season 2). *Note: N* = *Normal, H* = *Half, D* = *Double, Milo* = *Milorganite*.

Results of this analysis showed S_CO2_ emissions were significantly (p < 0.01) affected by organic amendments and application rates (Table 3). The analysis also showed all organic amendments and application rates had higher S_CO2_ emission rates than the control treatments (Table 3 and Figs. 6 and 7). Treatments with the highest chicken manure application rate had the largest S_CO2_ emission (167 kg ha^−1^ day^−1^-season 1, and 205 kg ha^−1^ day^−1^-season 2), and treatments with the lowest dairy amendment application rate had the lowest S_CO2_ emission (87 kg ha^−1^ day^−1^-season 1, and 101 kg ha^−1^ day^−1^-season 2). However, on average, season 2 had higher S_CO2_ emission than season 1, except for treatments with dairy at medium application rates. In compared to controls, S_CO2_ emission increased for each organic amendment and application rate (Fig. 7).

**Table 3:** Relationship of soil CO_2_ emission between control (ctrl, untreated) and organically amended plots.

Comparing soil CO_2_ flux from un-amended (Ctrl) and amended (applied three manures) plots										
	Ctrl	Chi-H	Chi-N	Chi-D	Dairy-H	Dairy-N	Dairy-D	Milo-H	Milo-N	Milo-D
**2017–2018: First Season**										
Mean	75	93	131	167	87	138	94	92	121	130
R^2^		0.26	0.42	0.17	0.67	0.16	0.27	0.46	0.46	0.24
p		0.0001	<0.0001	0.0045	<0.0001	0.0043	0.0001	<0.0001	<0.0001	0.0003
RMSE		27.8	24.8	29.6	18.7	29.8	27.8	23.8	23.8	28.3
**2018–2019: Second Season**										
Mean	94	114	168	205	101	119	117	124	152	164
R^2^		0.63	0.21	0.03	0.9	0.87	0.88	0.77	0.41	0.05
p		<0.0001	0.0003	0.1656	<0.0001	<0.0001	<0.0001	<0.0001	<0.0001	0.0667
RMSE		37.68	55.01	60.97	19.03	22.04	21.32	29.54	47.4	60.2

Unit of Mean is kg ha^−1^ day^−1^.

Growing season 1 showed the highest variability of S_CO2_ emission on plots treated with chicken at high application rate and lowest variability on controls (Fig. 7, Box and Whisker Plots). On the other hand, there was higher variability in S_CO2_ emissions for multiple amendments and their application rates during growing season 2. In comparison, all amendments and their application rates during the growing season 2 showed higher variability in S_CO2_ emissions than growing season 1; however, medium application rates had highest variability during growing season 2. In addition, season 2 showed higher mean, median and range of S_CO2_ emission compared to season 1. In season 2, all of the amended plots showed a higher range with median application rates, but their means and medians gradually increased with the increasing application rates. However, season 1 had completely different distributions for range, mean and median of S_CO2_ emission in compared to season 2 (Fig. 7). Soils amended with chicken manure had a higher range at high application rate, and mean and median gradually increased with the increasing amendment application rate, whereas soils amended with dairy manure showed higher range, mean, and medians of S_CO2_ emission at medium application rate. On the other hand, soils amended with milorganite showed the higher range and mean of S_CO2_ emission at high application rate but higher median at medium application rate.

Regression analysis showed a good correlation (R^2^ = 0.67) between S_CO2_ emission from controls and amended soils with dairy at a low application rate during the growing season 1. All other amendments and their application rates showed low correlation rates (R^2^ = 0.16 to 0.47); whereas S_CO2_ emission from multiple amendments and their application rates during the growing season 2 had high correlation rates with S_CO2_ emission than the control treatments. Carbon dioxide emission from all dairy treatments and low milorganite and chicken application rate treatments showed high correlations than the control treatments (R^2^ = 0.63 to 0.9) (Table 3). On the other hand, chicken and milorganite with high application rate treatments showed the least/lowest correlations (R^2^ = 0.03–0.05) with control treatments during growing season 2. In addition, soils amended with chicken and milorganite at high application rate treatments showed poor correlations and higher p-values (>0.01) with control treatments.

Table 4 compares N content for each organic amendments and application rates during two growing seasons and pH values at the end of the first growing season. Results showed the difference of N content between control and amended plots were small during both growing seasons. Control plots showed a slight decrease in N in the second growing season compared to the first growing season at both depths. The N content of the top and deep soil layers was 0.41 g N kg^−1^ during the first growing season, whereas those layers had 0.29 and 0.28 g N kg^−1^ during the second growing season, respectively. In comparison, none of the amended plots showed significant higher N content than control treatment plots during both growing seasons. However, in comparison, all of the amended plots showed higher nitrogen content than control treatment during the second growing season. The observed N contents of the topsoil layer amended with dairy manure and milorganite at were increased by the second growing season, however, N contents were decreased amended with chicken manure. On the other hand, all of the amended plots of the deep soil layer, except amended with milorganite at low application rates, showed lower N content by the second growing season.

**Table 4:** Nitrogen content (g kg^−1^) at two soil depths for three organic amendments and application rates in season 1 and 2. pH values at the at end of growing season 1.

	Growing Season 1		Growing Season 2		Change in N		pH	
	0–15 cm	16–30 cm	0–15 cm	16–30 cm	0–15 cm	16–30 cm	0–15 cm	16–30 cm
**Low Application Rate**								
Chicken	0.40	0.37	0.47	0.33	0.07	−0.04	5.94	5.37
Dairy	0.45	0.35	0.58	0.33	0.13	−0.02	6.18	5.37
Milorganite	0.36	0.29	0.52	0.45	0.16	0.16	6.14	5.3
Control	0.41	0.41	0.29	0.28	−0.12	−0.12	5.77	5.27
**Medium Application Rate**								
Chicken	0.56	0.41	0.48	0.37	−0.08	−0.04	5.64	5.26
Dairy	0.49	0.40	0.60	0.39	0.11	−0.01	6.47	5.39
Milorganite	0.41	0.38	0.64	0.36	0.23	−0.01	6.62	5.42
**High Application Rate**								
Chicken	0.57	0.48	0.52	0.36	−0.05	−0.13	6.04	5.57
Dairy	0.55	0.38	0.86	0.37	0.30	0.00	7.21	6.15
Milorganite	0.38	0.32	0.54	0.32	0.16	0.00	5.85	5.24

All of the amended plots including control treatments had higher pH at the topsoil layers than the deep soil layers. The plots amended with dairy manure and milorganite showed an increasing trend in pH with the increasing application rates at both soil layers except plots amended with milorganite which showed a decreasing trend from medium to a high application rate. However, plots treated with chicken manure showed a decreasing trend from low to medium application rate and increasing trend from medium to high application rate at both soil layers. Plots amended with dairy at high application rate showed the highest pH (slightly alkaline, 7.21) in the topsoil layer, whereas plots amended with milorganite at high application rate showed lowest pH (acidic, 5.24) in the deep soil layer.

Cumulative S_CO2_ emissions from all of the amended plots were higher than those from respective un-amended (controls) plots during both growing seasons (Fig. 6). However, season 2 had higher cumulative S_CO2_ emissions than season 1 from both amended and un-amended plots. For example, treatments of season 2 had 24% to 35% higher cumulative S_CO2_ emissions than those of season 1. This difference was only 5% higher during season 2 than season 1 for the dairy manure treatment at medium application rate. Soils amended with chicken manure at all application rates showed the highest cumulative S_CO2_ emissions in both seasons except for s amended with chicken at low application rate, which had lower cumulative S_CO2_ emission than soils amended with milorganite at low application rate during season 2. Similarly, soils amended with dairy at all application rates showed lowest cumulative emissions compared to milorganite and chicken amendments during both growing seasons except soil amended with milorganite at medium application rate, which had lower cumulative S_CO2_ emission than soils amended with dairy at medium application rate during growing season 1. Cumulative S_CO2_ emissions rates were directly proportional to the rate of application for all of the three treatments (Fig. 6).

### Relationships between soil CO_2_ emissions, total soil organic carbon, temperature and rain

Spatio-temporal variations of S_CO2_ emission are influenced by climatic parameters (e.g., temperature, and rain) and hydrologic parameters (e.g., soil moisture). The results showed rain and soil temperature influenced S_CO2_ emission during the growing seasons (Figs. 3 and 4). Relatively warmer air and soil temperatures, especially in April and May, resulted in higher S_CO2_ emission during the second growing season than the first growing season. Since air temperature influences soil temperature, seasonal variations in soil temperature rely on air temperature. However, although air temperature frequent had higher and lower peaks than the soil temperature, on average, both surface and deep soil temperatures were found warmer than the air temperature during the growing seasons, which might have impacted S_CO2_ emission. In addition, the combined effect of rainfall and warmer soil and air temperatures could explain the higher S_CO2_ emission during the second growing season.

Air and soil temperatures were almost consistently higher during the second growing season than those of the first growing season except in November and March. Monthly average air temperature in December 2017, January and February 2018 were 9.5, 6.2, and 13.6 °C, whereas temperatures of the same months during the second growing season (December 2018, January, and February 2019) were 12.2, 10.4, and 13.6 °C, respectively. However, surface and deep soil temperatures were higher in season 1 than season 2. Since the second growing season was warmer than the first growing season, higher S_CO2_ emission in season 2 indicates air and soil temperatures had more impact on S_CO2_ emission than the rainfall. In addition, the results showed an increase in S_CO2_ emission with the temperature rise from February to April (Figs. 3–5).

Control plots showed a decrease in total organic carbon (TOC) in the deep soil layer (16–30 cm) compared to the topsoil layer (0–15 cm) during both growing seasons. The TOC of top and deep soil layers were 3.35 and 3.24 g C kg^−1^ during the first growing season, whereas they had 2.67 and 2.62 g C kg^−1^ during the second growing season, respectively. In addition, TOC of the control treatment plots during the second growing season was lower than the first growing season. In contrast, the observed TOC was higher than that of the control treatment during the second growing season for most of the amended plots with different application rates. However, plots amended with chicken manure at high application rate at both soil layers, amended with dairy manure at low application rate at deep soil layer, amended with chicken manure and milorganite with medium application rates at deep soil layers had lower TOC during the second growing season than the first growing season (Table 5).

**Table 5:** Total soil organic carbon (g kg^−1^) at two soil depths for three organic amendments and application rates in season 1 and 2.

	Growing Season 1		Growing Season 2		Change in TOC	
	0–15 cm	16–30 cm	0–15 cm	16–30 cm	0–15 cm	16–30 cm
**Low Application Rate**						
Chicken	3.45 (0.10)	2.98 (−0.26)	4.23 (1.56)	3.01 (0.40)	0.78 (1.45)	0.03 (0.66)
Dairy	4.23 (0.89)	3.14 (−0.10)	6.11 (3.44)	3.04 (0.42)	1.88 (2.55)	−0.10 (0.52)
Milorganite	3.21 (−0.14)	2.80 (−0.44)	4.64 (1.97)	3.35 (0.74)	1.43 (2.10)	0.55 (1.17)
Control	3.35	3.24	2.67	2.62	−0.68	−0.63
**Medium Application Rate**						
Chicken	3.84 (0.49)	3.28 (0.04)	4.02 (1.35)	3.27 (0.65)	0.18 (0.86)	−0.02 (0.61)
Dairy	4.71 (1.37)	3.45 (0.21)	5.25 (2.58)	3.65 (1.03)	0.54 (1.21)	0.20 (0.82)
Milorganite	3.88 (0.54)	3.41 (0.17)	4.92 (2.25)	3.31 (0.70)	1.04 (1.72)	−0.10 (0.52)
**High Application Rate**						
Chicken	4.92 (1.58)	3.76 (0.51)	4.22 (1.55)	3.16 (0.54)	−0.71 (−0.03)	−0.60 (0.03)
Dairy	5.79 (2.44)	3.39 (0.15)	8.74 (6.07)	3.46 (0.84)	2.96 (3.63)	0.07 (0.70)
Milorganite	3.69 (0.35)	3.05 (−0.19)	5.35 (2.68)	3.08 (0.47)	1.66 (2.34)	0.03 (0.66)

The change in total organic carbon contents (g kg^−1^) with respect to the control are included inside the parentheses.

With respect to control treatments, between growing seasons 1 and 2, the highest increase in TOC (3.63 g C kg^−1^) was observed in the top (0–15 cm) soil layer of the dairy manure treatment with high application rates; whereas the lowest increase in TOC (0.03 g C kg^−1^) was observed in the deep (16–30 cm) soil layer of the treatments amended with chicken manure at high application rates. On the other hand, a small decrease of TOC (−0.03 g C kg^−1^) was observed in the topsoil layer amended with chicken manure at the high application rate.

## Discussion

The rate of S_CO2_ emission is dependent on many factors including environmental conditions, soil characteristics, and land covers; however, it primarily depends on the application rates and types of organic amendments. In this study, soil carbon dioxide emissions under collard greens were studied in response to organic amendment type and rate in addition to rainfall and soil temperature. In general, carbon dioxide emission increased with increased soil temperature and following rainfall events; this response varied spatially and temporarily because the spatio-temporal variability of soil temperature and moisture might have altered the spatial and temporal distributions of soil organic matter or soil organic carbon; these findings concur with the findings of Junior *et al*.^48^. The observed increase in S_CO2_ emission in response to increasing temperature and soil moisture/rainfall indicate an increase in microbiological activities; such activities result in an increase of the soil organic carbon and also carbon dioxide emission because S_CO2_ is mainly produced as a result of the soil microbial respiration and vegetation root respiration^48–50^.

Growing Season 2 was warmer than growing season 1, but both seasons had similar cumulative rainfall. Therefore, although the soil temperature and soil moisture/rainfall have been identified as the most critical environmental factors affecting S_CO2_ emissions, results showed only soil temperature had a sufficiently great impact on S_CO2_ emissions, result in higher S_CO2_ emissions in growing season 2 than growing season 1. These findings concur with the previous studies^42,51,52^. In addition, both growing seasons showed a gradual decrease in S_CO2_ emissions during the period of low soil temperature (November-February).

Since the carbon content of dairy manure was the lowest among the three, the observed rate and cumulative S_CO2_ emissions from the dairy amended plots were low during both growing seasons. Moreover, both chicken manure and milorganite had more nitrogen (N), phosphorus (P) and potassium (K) than dairy manure. On the other hand, chicken manure contained less N, but more P and K than milorganite. In addition, the application rates (low, medium, and high) were directly proportional to the S_CO2_ emissions because the amount of applied carbons and N, P, K to the soil were increased accordingly. Also, observed cumulative S_CO2_ emissions from soils amended with chicken manure were high even though milorganite had more carbon content, indicating that chicken manure treatments might enhance microbial activities more that could be the result of their higher content of P and K than milorganite. Hence, there might be different elements, e.g., C, N, P, and K available in organic amendments responsible for these higher S_CO2_ emissions. In addition, there might be a combined effect of these elements available in organic amendments and nitrogen, pH (measured), electrical conductivity (EC), K, P, etc. (not measured) available in the soil, and climatic variables. However, it is also true that organic amendments having higher carbon and nitrogen, and lower pH would most likely enhance S_CO2_ emissions^53^.

We found S_CO2_ emissions from soils amended with dairy at a low application rate in growing season 1 and all application rates in growing season 2 significantly correlated with the S_CO2_ emissions from the un-amended soils (R^2^ = 0.67 to 0.9). On the other hand, soils amended with chicken manure and milorganite with all application rates were poorly correlated with the S_CO2_ emissions from the un-amended soils in both seasons except amended with milorganite and chicken at low application rates in growing season 2. These correlations indicate that the amount of carbon contents in respective organic amendments had a significant impact on S_CO2_ emissions.

Dairy manure treatments had the highest increase in total soil organic carbon (up to 3.63 g C kg^−1^ at high application rate) by the end of growing season 2, indicating that dairy manure might have higher potential of soil carbon sequestration for the shallow soil layer and a low potential of soil carbon sequestration for the deep soil layer. On the other hand, even though carbon and nitrogen contents of milorganite and chicken manure were high, an increase in total organic carbon by the end of the growing season 2 was low. Therefore, it shows that carbon and nitrogen contents of organic amendments are not only the controlling factors to impact the soil organic carbon, but there are other controlling factors such as soil water management, climatic and environmental combined with the carbon, nitrogen, and other nutrients that affect total soil organic carbon.

Overall, it is worth noticing that the response of S_CO2_ emission to organic amendment types and their application rates are also dependent on environmental factors such as temperature, rainfall, and soil characteristics, and other management factors.

## Conclusions

It is important to understand the impact of organic amendments and application rates on S_CO2_ emissions under crop production. This study used three organic amendments and three application rates to understand the impact of S_CO2_ emission, change in total organic content of the soil under a collard greens crop in the southeast Texas environment during two growing seasons.

There was a significant impact of organic amendment types and application rates on S_CO2_ emissions and total soil organic carbon. More cumulative CO_2_ was emitted from chicken manure treatment followed by milorganite and then dairy manure. Carbon dioxide emission was affected more by the applied organic amendments and application rates than by air/soil temperature and rainfall during the two growing seasons.

While dairy manure with lowest carbon content (C) and N, P, K showed the least cumulative S_CO2_ emission, chicken manure with highest P, K but lower C and N than milorganite showed the highest cumulative S_CO2_ emission in both growing seasons. However, on average, TOC increase was the highest in growing season 2 in soils amended with dairy manure. Therefore, TOC content of soils and rate of S_CO2_ emissions, which help in soil carbon sequestration would highly depend on the types and rates of applied organic amendments and local environmental conditions including soil temperatures, amount and frequency of rainfall as well as soil characteristics (e.g., texture, organic matter content, and porosity).

Overall, this study helps to develop best management practices to maximize carbon sequestration and to minimize S_CO2_ emissions from agricultural soils, not only for collard greens, but also for other crops grown in the southeast Texas and eventually for the entire southeast region of the U.S. Further, it is recommended to evaluate the impact of organic amendment types and application rates on the yield of collard greens. Since S_CO2_ emission rate was extremely high after the application of the organic amendment, it might be better to incorporate the organic amendments slightly deeper into the soil profile to minimize their losses.
